# Transverse myelitis as a rare paraneoplastic manifestation of a pancreatic tail adenocarcinoma: A case report

**DOI:** 10.1097/MD.0000000000042133

**Published:** 2025-05-23

**Authors:** Ethan Burg, Gabrielle Sharbin, Max E. Edeson, Hanieh K. Hosseini, Colin Westman, Davood K. Hosseini, Shil Patel, Jonathan D. Weinberger, Hongfa Zhu, Rosario Ligresti

**Affiliations:** a Hackensack Meridian School of Medicine, Nutley, NJ; b Thomas J. Long School of Pharmacy, Stockton, CA; c Department of Gastroenterology, Palisades Medical Center, Hackensack, NJ; d Department of Gastroenterology, Hackensack University Medical Center, Hackensack, NJ; e Department of Pathology, Hackensack University Medical Center, Hackensack, NJ.

**Keywords:** case report, pancreatic tail adenocarcinoma, paraneoplastic syndrome, transverse myelitis

## Abstract

**Rationale:**

Transverse myelitis (TM) is a non-compressive myelopathy caused by spinal cord inflammation that can present with motor, sensory and autonomic deficits. Paraneoplastic TM represents a rare etiology of TM caused by autoantibodies targeting the spinal cord in the setting of malignancy, most frequently lung cancers and lymphoproliferative disorders. Anti-glutamic acid decarboxylase 65 (GAD65) is a rare antibody associated with paraneoplastic neurological sequelae. This case presents a GAD65 positive paraneoplastic TM in the setting of a pancreatic tail adenocarcinoma.

**Patient concerns:**

A 51-year-old male with an 18.5 pack year smoking history presented for 4 months of progressively worsening left-sided weakness and back pain. He endorsed numbness, tremors and paresthesias of his left upper extremity, left lower extremity weakness with unsteady gait.

**Diagnoses:**

Magnetic resonance imaging demonstrated C4-T1 demyelination consistent with TM. The serum paraneoplastic panel revealed a positive GAD65 prompting further workup for occult malignancy. Computed tomography chest abdomen and pelvis and magnetic resonance imaging with magnetic resonance cholangiopancreatography showed pancreatic body and tail enlargement. A pancreatic tail mass was confirmed on endoscopic ultrasound. Pathology was consistent with pancreatic ductal adenocarcinoma, confirming the diagnosis of a paraneoplastic TM.

**Interventions:**

He received 5 days of methylprednisolone (1.0 g daily) and plasmapheresis for initial treatment of his TM and 5 days of methylprednisolone for his repeat episode. He was being treated for stage III T4N1 pancreatic cancer. He had completed 2 cycles of oxaliplatin, irinotecan, leucovorin, and 5-flurouracil (mFOLFIRINOX).

**Outcomes:**

The patient initially had improvement in his neurological symptoms following the first chemotherapy cycle but developed quadriparesis requiring readmission for subacute TM. He transitioned to hospice care given progression of malignancy.

**Lessons:**

This case highlights the importance of GAD65 as a paraneoplastic antibody and promotes awareness of pancreatic tail adenocarcinoma as a rare cause of paraneoplastic TM.

## 1. Introduction

Myelopathies, or spinal cord compression, can be compressive or noncompressive.^[1]^ Transverse myelitis (TM) is a rare form of non-compressive myelopathy due to spinal cord inflammation,^[1–4]^ affecting 1 to 8 individuals per million annually.^[3–6]^ TM symptoms vary depending on the spinal cord level involved. Patients can present with motor/sensory deficits and autonomic impairment below the level of the lesion.^[2]^

Etiologies of TM can be classified as parainfectious, paraneoplastic, drug/toxin induced, autoimmune and acquired demyelinating diseases.^[2]^ There are also noninflammatory etiologies that can mimic TM’s presentation, most commonly idiopathic.^[2,3]^

Paraneoplastic myelopathy is a rare manifestation of malignancy that can cause cord necrosis presenting as an ascending transverse lesion or patchy multifocal necrosis, involving spinal cord white matter.^[7,8]^ It is hypothesized that the malignancy and nervous system share antigens resulting in an autoimmune response.^[7,8]^ Antibodies targeting voltage gated calcium/potassium channels, amphiphysin, ganglionic acetylcholine receptors, and anti-neuronal nuclear antibodies, have been associated with paraneoplastic spinal cord lesions.^[7,9]^ Paraneoplastic myelopathies generally develop rapidly and are severe, with few patients improving following treatment.^[7,10,11]^ Magnetic resonance imaging (MRI) findings may include T2-weighted signal abnormalities in a longitudinal tract/gray matter distribution, with symmetric enhancement.^[7,9,11]^ Patchy gadolinium enhancement can also be seen in necrotizing paraneoplastic myelopathy.^[11]^ Cerebrospinal fluid analysis may show increased protein.^[7]^ Paraneoplastic TM has mainly been associated with pulmonary and lymphoproliferative malignancies.^[7]^ We present a rare case of paraneoplastic TM in the setting of pancreatic tail adenocarcinoma.

## 2. Case report

A 51-year-old unemployed, African American male with a history of hypertension, hyperlipidemia, obesity and a tobacco smoking history with 18.5 total pack years, presented to the emergency department for 4 months of progressively worsening left-sided weakness and back pain. The patient endorsed numbness, tremors and paresthesias of his left upper extremity and an unsteady gait due to left lower extremity weakness. He denied urinary/bowel incontinence and fever. He was seen 1 month prior for similar symptoms and underwent lumbar and cervical MRI which showed degenerative changes of the spine, but was otherwise unremarkable. Physical exam was significant for ⅖ left upper and lower extremity weakness with abnormal gait. He had frequent stimulus-induced myoclonic spasms of the left hand and leg with brisk reflexes throughout and positive bilateral Hoffmann and Babinski reflexes. Sensation to light touch was intact throughout.

**Figure 1.**
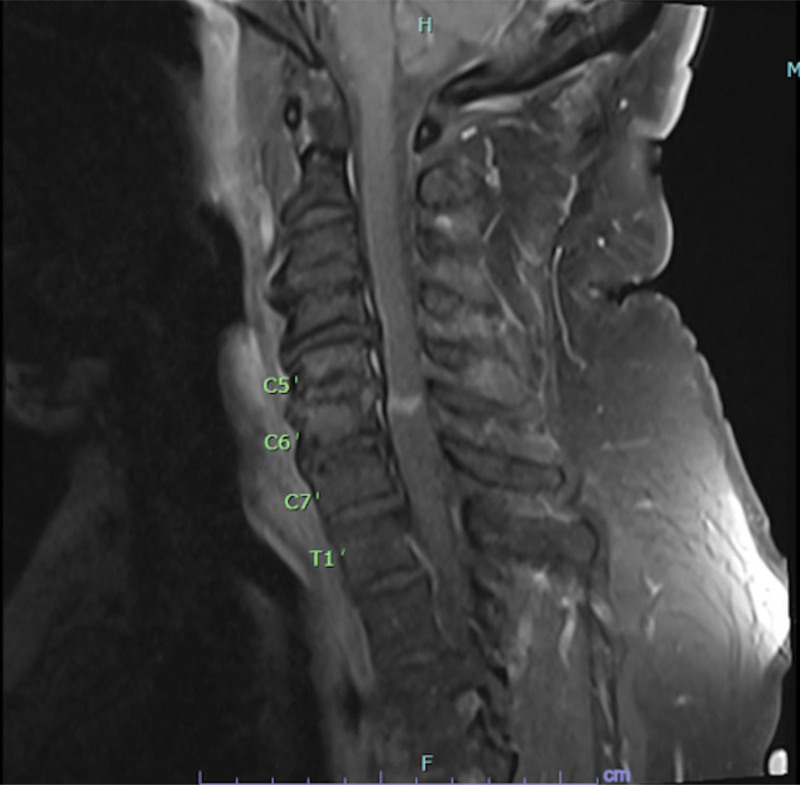
Magnetic resonance imaging of cervical spine with and without contrast showing acute demyelination consistent with transverse myelitis.

**Figure 2.**
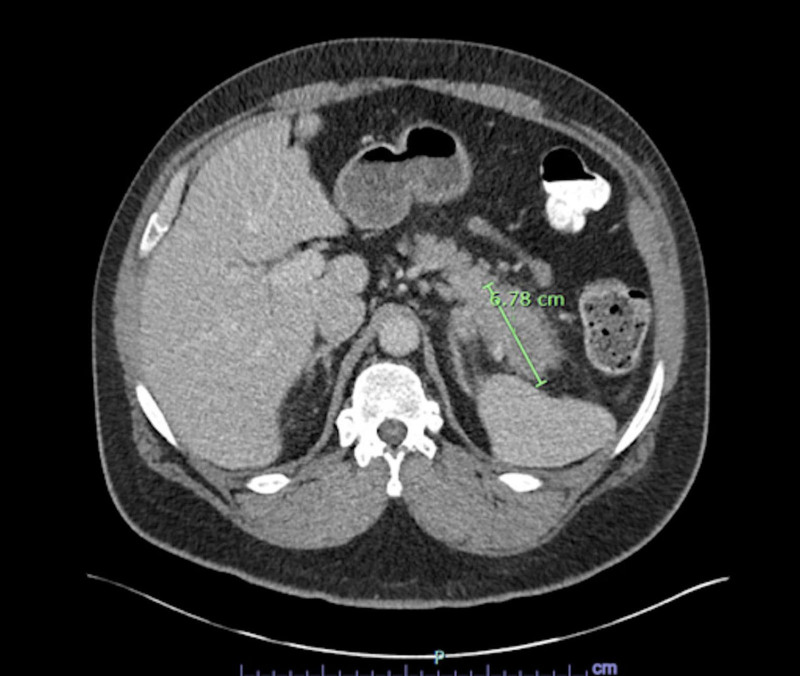
Computed tomography of the abdomen with contrast showing ill-defined mass-like thickening of the pancreatic tail.

**Figure 3.**
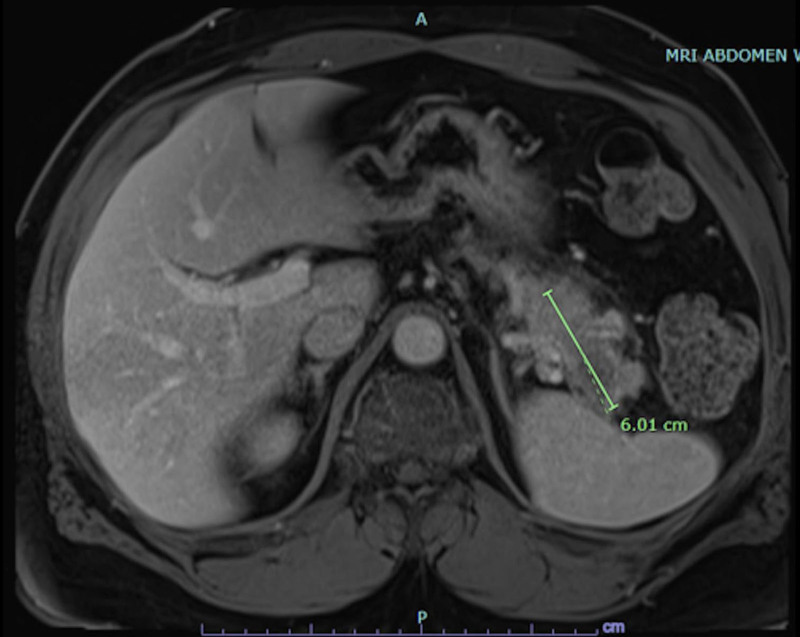
Magnetic resonance imaging of the abdomen with and without contrast showing segmental enlargement of the pancreatic tail.

MRI of the cervical spine with and without contrast showed evidence of acute demyelination consistent with TM (Fig. 1). Cerebrospinal fluid (CSF) studies were significant for elevated protein 89.9 mg/dL and Immunoglobulin G (IgG) index 1.38 in line with TM. CSF glucose was elevated 132 mg/dL as well as albumin 67.4 mg/dL, however white blood cells measured 0 cells/mm^3^. Additional CSF studies for conditions such as multiple sclerosis (MS) and neurosarcoidosis were negative with a negative myelin basic protein < 2 mcg/L and angiotensin converting enzyme 7 U/L. Infectious CSF studies were also unrevealing with Venereal Disease Research Laboratory testing, Lyme disease IgG and immunoglobulin M (IgM) antibodies and meningitis film array all being negative. Serum human immunodeficiency virus antigen/antibody and west nile virus IgM/IgG were not detected. Serum autoantibody testing for inflammatory demyelinating disease besides MS including antinuclear antibody, neuromyelitis optica/aqua porin 5 (NMO/AQP4) and myelin oligodendrocyte glycoprotein were negative. The patient’s vitamin B12 level was also normal (641 pg/mL). He received 5 days of high dose steroids (methylprednisolone 1 g daily) and plasmapheresis for treatment of TM. With no clear identifiable cause of his TM from prior studies, a serum paraneoplastic panel was sent for further work up revealing positive anti-glutamic acid decarboxylase 65 (GAD65) at 0.04 nmol/L (reference < .02), prompting further imaging for malignancy. Computed tomography (CT) chest abdomen and pelvis with contrast showed an ill-defined mass-like thickening of the pancreatic tail measuring 6.8 cm (Fig. 2). MRI abdomen with and without contrast and magnetic resonance cholangiopancreatography showed segmental enlargement/interstitial edema of the distal pancreatic body and tail spanning 6 cm (Fig. 3). Serum carcinoembryonic antigen and carbohydrate antigen 19-9 were elevated to 28.7 ng/mL and 4024 U/mL respectively.

**Figure 4.**
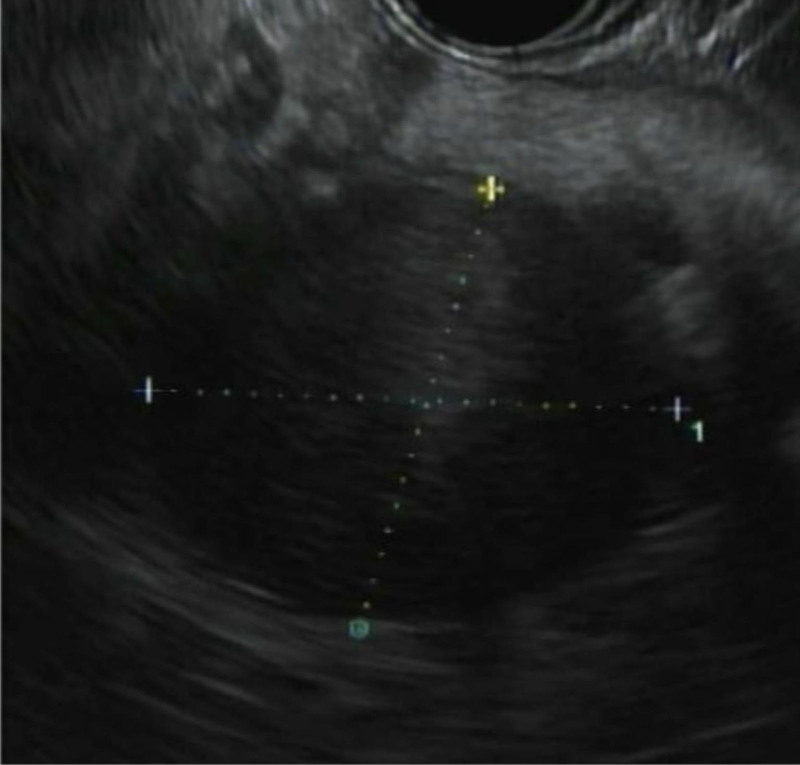
Endoscopic ultrasound of 28 × 40 mm hypoechoic pancreatic tail mass.

**Figure 5.**
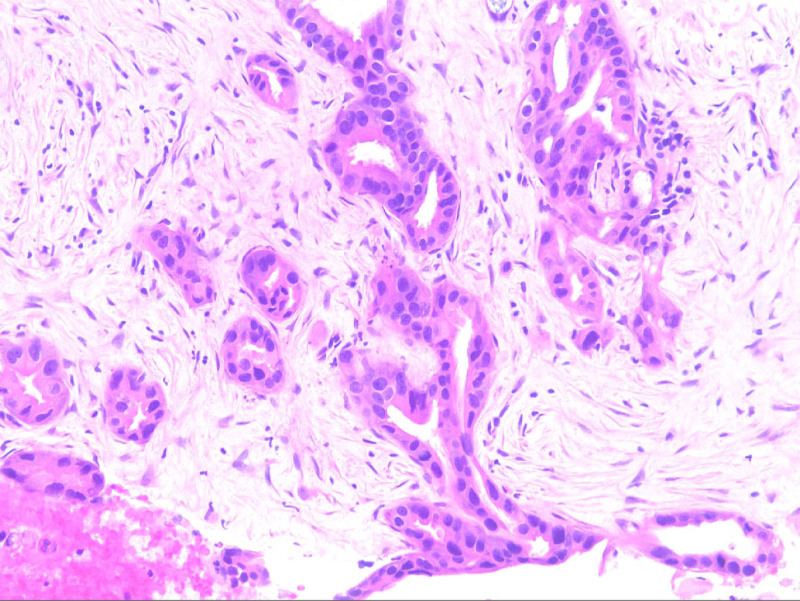
Pancreatic ductal adenocarcinoma. Hematoxylin and Eosin stain x200.

He underwent endoscopic ultrasound with biopsy which showed a 28 × 40 mm hypoechoic mass in the pancreatic tail with scattered 1 cm peripancreatic lymphadenopathy and concern for splenic artery and vein invasion (Fig. 4). Histopathology was consistent with pancreatic ductal adenocarcinoma (Fig. 5). Given the patient’s pancreatic cancer and GAD65 paraneoplastic antibodies with otherwise negative studies for other possible diagnoses, it was determined that this was the most likely cause of his TM.

The patient was discharged and was undergoing treatment for stage III T4N1 pancreatic cancer. He completed 2 of 6 total cycles of oxaliplatin, irinotecan, leucovorin, and 5-flurouracil (mFOLFIRINOX) with initial mild improvement in his mobility and back pain after the first cycle. However, after this initial improvement his mobility and back pain continued to decline, progressing to quadriparesis requiring admission. Repeat MRI cervical, thoracic and lumbar spine showed findings consistent with subacute TM through the thoracic cord at the level of C6. He was subsequently treated with a repeat 5 day course of 1.0 g of methylprednisolone daily with only mild improvement in finger flexion strength. During this repeat admission the patient was found to have new bilateral pulmonary embolisms and progression of his cancer with new hepatic and pulmonary lesions on CT chest abdomen and pelvis. Given the patient’s poor prognosis he was ultimately transitioned to hospice care where he ultimately passed.

## 3. Discussion

Paraneoplastic TM is a rare entity usually associated with lung and lymphoproliferative disorders.^[7]^ To date there have been no reported cases of TM in the setting of pancreatic adenocarcinoma.

Moreover, this represents the first case of GAD65 associated TM in pancreatic adenocarcinoma. GAD65 is rarely associated with paraneoplastic TM.^[12,13]^ It is more frequently found in patients with non-neurological autoimmune diseases.^[12]^ Neurological autoimmune conditions linked to GAD65 include conditions such as stiff person syndrome and limbic encephalitis.^[12]^ The few malignancies associated with GAD65 have been lung/breast cancers and thymomas.^[13]^

Patients with GAD65-associated neurological diseases are rarely associated with occult malignancy.^[12,13]^ In patients with positive GAD65 antibody and classic paraneoplastic syndrome (PNS) neurological presentations such as TM, the risk of underlying cancer is 10-fold higher. This risk is positively correlated with higher GAD65 titers.^[13]^ Patients with neuronal cell surface antigens (GABA receptors) in addition to GAD65 had a 7-fold increased risk of malignancy.^[13]^

There are no formal recommendations regarding the oncological work up of suspected PNS in the setting of GAD65. However, given the association of both neurological PNS and GAD65 neurological PNS to lung cancer, patients should undergo CT/positron emission tomography-CT chest.^[13]^ Whole body PET-CT imaging may be considered with additional neoplastic workup.^[13]^ Patients with a concern for pancreatic malignancy should undergo appropriate abdominal imaging and advanced testing i.e. endoscopic ultrasound, CT/MRI abdomen and pelvis for diagnosis.

PNS TM is difficult to treat, but treatment of the underlying malignancy may lead to improvement or stabilization of symptoms. If there is a lack of improvement then an immunotherapy trial may be appropriate.^[12]^

## 4. Conclusion

The detection of pancreatic tail adenocarcinoma is difficult due to the late onset of clinical features leading to diagnosis at an advanced stage. Pancreatic malignancies should be considered in patients with suspected PNS, specifically those with GAD65 antibodies, and may provide an opportunity for earlier detection.

## Author contributions

**Conceptualization:** Ethan Burg, Gabrielle Sharbin, Max E. Edeson, Hanieh K. Hosseini, Davood K. Hosseini, Jonathan D. Weinberger, Rosario Ligresti.

**Data curation:** Max E. Edeson, Hanieh K. Hosseini, Colin Westman, Davood K. Hosseini, Shil Patel, Jonathan D. Weinberger, Hongfa Zhu.

**Investigation:** Davood K. Hosseini.

**Methodology:** Davood K. Hosseini.

**Writing – original draft:** Ethan Burg, Gabrielle Sharbin, Max E. Edeson, Hanieh K. Hosseini, Davood K. Hosseini, Rosario Ligresti.
